# Heliotropism

**Published:** 1910-08-06

**Authors:** 


### Heliotropism.

There is unfortunately a fashion in scientific theories just as much as there is a fashion in ladies' hats, and a theory is not rejected because it can be demonstrated fundamentally erroneous, but simply because another comes which is more interesting. It is then customary to speak with patronising contempt of the efforts of thinkers before modern inventions made all things clear, though no one takes the trouble to notice how closely the ancient and rejected theory is allied to the modern and approved. Few even have taken the trouble to find out what the earlier writer did really mean. Generally he is quoted simply as not having known what we now are supposed to know. According to modern ideas, and according to the most humble text-book on botany and zoology, the sun is the author of energy, and so far as concerns this world the author of life. The ancient sun-worshippers in their pitiful blindness must actually have stumbled upon a scientific truth which in our enlightened days we regard as fundamental. Few, however, have realised how all-important this responsiveness to sunlight must be. It has been noticed even with unicellular animalcules that they are to a certain degree heliotropic, which quality increases as we go up the scale. It is not to be assumed that what is pleasant to an animal is *ipso facto* healthy, but it is certainly not incorrect to suppose that in the case of basking in the sun, a habit indulged in by so many animals, be they reptiles, birds, or mammals, is not without its significance in affecting their constitution as a whole. The sun's rays are now employed therapeutically, and, from the human standpoint, the effects of sunlight have a singular psychological intensity. There is a quickening and enlivening effect, which on days of perfect weather makes itself noticeable by a peculiar sense of elation. Probably at the bottom of our ideas of the beautiful lies the sun's influence. Multitudinous treatises have naturally been written to explain the origin of our ideas of æsthetics, and many have postulated various innate virtues which, being worthy of man's exalted position, are inseparably connected with our appreciation of the beautiful. It is, of course, quite clear that without the light we could not see, but the point is that the exhilarating effect of sunlight produces in us a feeling of satisfaction with our surroundings, from which arises, in a rudimentary way, our notion of the beautiful. Consider, for example, the effect of artificial light and scenery through such a medium as that of the stage. For the majority of the audience the greatest pleasure is experienced when the imitation approaches nearest to Nature. The glare of artificial lights, such as can be seen any time in the large thoroughfares of London, does not produce a beneficial feeling. The attraction of the moth to the lamp more nearly expresses the nature of the psychological effects of artificial light, but the reason for the moth's attraction still remains obscure. It is possible that a sense of the beautiful is intimately connected with the higher development of man on principles of health, and that those who are really unable to appreciate natural beauties are abnormal, or at least wanting in development. The contrasts of light and shade, which appeal to us so much while walking through a wood, and appeal with lessened force when represented on canvas, are no less potent when described in the language of some fine poet. Most of Pierre Loti's wonderful effects are produced in all his books by his clear insight into this helic- or photo-trophism manifested by the animal.

